# Exploring healthcare workers’ immunisation behaviour towards COVID-19 vaccines through psychological patterns

**DOI:** 10.4102/phcfm.v17i1.4710

**Published:** 2025-01-24

**Authors:** Nour El Houda Benkaddour, Sara Ramdani, Hind Khalil, Asmae Lekfif, Naima Abda, Bouchra Oneib, Yassamine Bentata

**Affiliations:** 1 Laboratory of Epidemiology, Clinical Research and Public Health, Faculty of Medicine and Pharmacy, Mohammed First University of Oujda, Oujda, Morocco; 2 Maternal-Infant and Mental Health Research Laboratory, Faculty of Medicine and Pharmacy, Mohammed First University of Oujda, Oujda, Morocco; 3 Department of Psychiatry, Mohammed VI University Hospital, Oujda, Morocco; 4 Nephrology and Kidney Transplantation Unit, Mohammed VI University Hospital, Oujda, Morocco

**Keywords:** vaccination behaviour, stress, vaccination perception, COVID-19, healthcare workers, Morocco

## Abstract

**Background:**

The psychological approach can provide valuable insights into vaccination behaviour, especially in high-risk contexts. It offers new perspectives for effective interventions to improve vaccination behaviour.

**Aim:**

To investigate key factors influencing stress related to vaccination in emergency situations among healthcare professionals.

**Setting:**

Eastern region of Morocco.

**Methods:**

We conducted a descriptive and analytical cross-sectional study involving 221 healthcare professionals in the Eastern region of Morocco. A snowball sampling method was used to select the participants who were administered a questionnaire. Logistic regression analysis was performed with *p* < 0.05 being the level of statistical significance.

**Results:**

The participants had a median age of 25.5 years (30–34.5) and were predominantly females (68.3%). Vaccination coverage stood at 84.6%, with a positive perception of 77.8%. The analysis of the Perceived Stress Scale (PSS) revealed that 51.6% (*n* = 114) of healthcare professionals experienced stress towards vaccination. Females were almost two times more susceptible to experiencing vaccination stress (*p* = 0.03). Furthermore, the analysis showed that vaccination profile (*p* = 0.02), accepting the vaccine for any reason other than its accessibility (*p* = 0.03) and having a previous coronavirus disease 2019 infection (*p* = 0.03), were significantly associated with stress. In contrast, healthcare professionals based at the university hospital had a significantly lower stress level (*p* = 0.01).

**Conclusion:**

Moroccan healthcare professionals exhibited high vaccine acceptance and positive perceptions, particularly among vaccinated individuals despite notable stress around immunisation.

**Contribution:**

These insights can guide governments and policymakers in developing strategies to enhance healthcare workers’ awareness and understanding of vaccination.

## Introduction

Throughout human history, numerous pandemics have occurred, and it is because of the concept of vaccination that entire populations have not been decimated. However, as Brewer et al. suggest, the great success of immunisation programmes has left many people unaware of, and unconcerned about, diseases they have never encountered.^1^ This lack of awareness, combined with unsubstantiated safety concerns frequently amplified in traditional and social media, can create doubt about the effectiveness of vaccines. As a result, even though vaccines remain a critical tool in preventing disease, public confidence in them can waver.

Despite its long history, the issue of reticence and resistance towards vaccination has been intensified over the past few decades, particularly during the development of vaccines against Severe acute respiratory syndrome coronavirus 2, the causative agent of the coronavirus disease 2019 (COVID-19).^2,3,4^ Consequently, a behavioural ecosystem has emerged around vaccines.^5,6^ We observe that immunisation yields the promised benefits when the majority of individuals adhere to vaccine recommendations.^7^ However, this progress is hindered by the conceptual dilemma of vaccine acceptance behaviour, which constrains the advancement of immunisation campaigns.

The accelerated development and rapid approval of the SARS-CoV-2 vaccines remain debatable among healthcare professionals, aiming to ensure increased security. This has a significant impact on public decision-making.^8,9^ Previous studies have underscored the importance of healthcare professionals’ opinions on vaccination coverage. For instance, a study in the United States using data from the National Immunization Survey-Teen (2010–2011) examined how healthcare provider recommendations affect HPV vaccine initiation among adolescent males aged 13–17 years. The results showed that adolescents whose parents received a healthcare provider’s recommendation were significantly more likely to begin the vaccine series, with an odds ratio of 19.02. In contrast, those whose parents did not receive such a recommendation had much lower vaccination rates. This study highlights the critical role of healthcare professionals in promoting vaccine uptake and underscores how their recommendations are crucial to the success of vaccination campaigns.^10,11^

Additionally, psychological science has the potential to offer significant insights into immunisation behaviour. Indeed, vaccination holds considerable benefits at both the individual and collective levels, particularly from a psychological perspective. The act of getting vaccinated is driven by a strong intention and a deliberate decision-making process. Within the framework of behavioural science, the concept of motivation is central to various psychological models.^1,12^ Motivation, which drives vaccination behaviour, is closely influenced by intention. Consequently, it is no coincidence that numerous studies focus on the role of thoughts and emotions in motivating individuals to get vaccinated.^1,12^ Furthermore, and given the increased eco-epidemiological risk, psychological sciences may provide new impetus for potential effective interventions in vaccination behaviour.^1^

The aim of this study was to examine the major factors influencing stress related to vaccination in emergency situations among a population of healthcare professionals.

## Research methods and design

### Study design

A descriptive and analytical cross-sectional study was conducted from February 2022 to November 2022. The survey was undertaken among medical and paramedical staff in the Eastern region of Morocco, using a face-to-face questionnaire administered in French.

### Setting

The study was conducted among medical and paramedical staff working in various healthcare facilities in the Eastern region of Morocco. These included Mohammed VI University Hospital, Regional Hospital Centre Al Farabi of Oujda, Provincial Hospital Centre Eddarak of Berkane, Provincial Hospital Centre Hassani of Nador as well as primary healthcare centres across the region.

### Study population

The study population comprises of medical and paramedical staff working in hospitals in Morocco. This group comprised general practitioners, doctors in specialty training, specialists, supervisors, nurses and health technicians.

The criteria of inclusion in the study were: being medical or paramedical staff working in the hospitals of the Eastern region in Morocco, being of ≥ 18 years of age and irrespective of years of experience. Staff who refused to participate were not included in the study.

### Sample

Sample calculation was performed online with the free and open-source software: ‘Open Source Statistiques Épidémiologiques pour la Santé Publique’.^13^

For an error risk α = 2%, an estimated proportion of positive perception: *P* = 99%,^14^ and a population size: *N* = 2493 health professionals, in the Eastern region of Morocco, the minimum required number of participants for the present study is 184.

In this study, the selection of the participants was conducted using a snowball sampling method, which was particularly suited for reaching healthcare professionals.^15^

Initially, we identified a small group of healthcare workers who met the study’s criteria and distributed the survey questionnaire to them. These initial participants were then asked to refer other healthcare professionals with similar characteristics. This process continued as referrals were made, allowing us to expand our sample within the healthcare sector. This method facilitated access to a network of professionals who might otherwise have been difficult to reach, thus providing a more comprehensive view of the target population.

### Data collection

The questionnaire used in our study was specifically developed by the authors to address the unique needs and objectives of our research. It consists of nine parts. Part one describes the survey and its objectives, including the voluntary, anonymous and confidential nature of the questionnaire. The second part begins with a question on consent and covers demographic data such as sex, age, marital status, workplace location, occupational status, educational level, work’s department, information on respondent’s seniority and chronic disease investigations. Section three assesses perceptions of COVID-19, compliance with sanitary measures and source of information about the disease. The fourth part presents data specific to SARS-CoV-2 vaccination, notably the vaccination profile of healthcare workers. Section five examines the perception of immunisation against SARS-CoV-2 infection, including the perception of vaccines recommended, seasonal influenza immunisation profile and recommendations for vaccination before and after receiving the vaccine. Section six is reserved for providing information about the COVID-19 disease, including a personal antecedent of COVID-19.

The seventh part is devoted to the assessment of anxiety via the Zung Anxiety Self-Assessment Scale (SAS).^16^ The SAS was designed by Zung to quantify a patient’s level of anxiety. It is a 20-item self-report tool designed to measure anxiety levels; each item is scored on a 1-to-4-point Likert-type scale according to these ratings: ‘Rarely’, ‘Sometimes’, ‘Often’ and ‘Almost always’. Overall scores can range from 20 to 80. A total score between 20 and 44 indicates normal anxiety, between 45 and 59 anxiety ranges from mild to moderate, between 60 and 74 anxiety is considered severe, while a score above 75 indicates severe to extreme anxiety.

The eighth part measures the level of perceived stress. The Perceived Stress Scale (PSS) is a classic instrument to evaluate perceived stress.^17^ The 10-item PSS self-questionnaire invites the participant to rate each element on how often it appears within a given period, on a 5-point scale ranging from 0 = never to 5 = very often, with a total score varying between 0 and 40. Scores from 0 to 13 are deemed to indicate low stress. From 14 to 26, stress level is considered moderate. A score of 27 to 40 is classed as high perceived stress.

The ninth part measures the severity of insomnia, using the Insomnia Severity Index (ISI).^18^ This brief 7-item scale is used to determine the type of insomnia by evaluating the individual’s sleep experience, daily functioning and thus their anxiety related to sleep disorders.

### Statistical analysis

We expressed quantitative variables as median and interquartile range, while categorical variables were expressed as percentages.

The Chi-square (χ^2^) test or Fisher’s exact test was used to compare categorical variables, as all variables in this study were categorical and did not include any quantitative variables. A *p*-value of less than 0.05 was considered statistically significant. Variables that met the significance threshold of *p* ≤ 0.05 were included in the backward stepwise logistic regression model, with the variable ‘vaccination profile’ included as a forced variable. The backward stepwise logistic regression method was chosen because of the large number of explanatory variables and the need to simplify the complex model by focusing on the most significant predictors. This approach allowed us to control for potential confounding factors and assess the independent effect of each explanatory variable on the variable of interest. The IBM SPSS Statistics software, Version 21.0, supported all statistical analyses.

### Ethical considerations

The protocol of this study was examined and approved by the Ethics Review Committee for Biomedical Research of the Faculty of Medicine and Pharmacy of Oujda (CERBO) according to the guidelines of the Helsinki Declaration. The submission file is registered and approved under the order number: 44/2021.

Prior to the start of the survey, written consent was obtained from each participant. Respondents were informed about the aims of the study, its voluntary nature and therefore the anonymity and confidentiality of the data.

## Results

### Sociodemographic characteristics of the participants

Two hundred and twenty-one copies were gathered from 250 distributed, resulting in a response rate of 88.4%, most of which were from the Mohammed VI University Hospital Centre (CHU Mohammed VI).

Around two-thirds of our participants were females (68.3%; *n* = 151), whereas males represented the remaining one-third (31.7%; *n* = 70), female to male ratio of 2.1. The median age of the participants was 25.5 years (30–34.5).

Among the 221 included in this survey, 49.8% (*n* = 110) were nurses, 26.7% (*n* = 59) were training residents, 16.3% (*n* = 36) were laboratory technicians, 3.6% (*n* = 8) were specialist physicians, 2.3% (*n* = 5) were general physicians and 1.4% (*n* = 3) were specialist physicians and university professors.

In terms of the distribution of healthcare professionals included according to the most common departments, 19.9% (*n* = 44) of them worked in the laboratory, 10.4% (*n* = 23) in neonatology, 8.1% (*n* = 18) in psychiatry, 7.7% (*n* = 17) in nephrology and 5.9% (*n* = 13) in the department of anaesthesia and intensive care.

### Vaccination profile and perception of coronavirus disease 2019 vaccination

Vaccination coverage in the sample was rated at 84.6% (*n* = 187), of which 59.4% (*n* = 111) developed adverse events. Vaccinated participants who received the third dose of the vaccine, represented 42.8% (*n* = 80) of all immunised respondents. However, 15.4% (*n* = 34) did not receive any vaccine and are considered unvaccinated.

The positive perception of SARS CoV-2 vaccines decreased from 77.8% (*n* = 172) for the first two doses to 43.4% (*n* = 96) for the third dose. The population of vaccinated subjects expressed a better perception of the vaccine doses, with a positive perception for the 1st and 2nd doses of 90.4% (*n* = 169) and 51.3% (*n* = 96) for the 3rd dose. However, this perception of the first of the two doses did not exceed 10% for the non-vaccinated population (8.8%; *n* = 3) and who were all against the third dose.

Fear of eventual side effects was the most frequent reason cited for non-acceptance or hesitation regarding vaccination against COVID-19 (73.5%; *n* = 25). On the other hand, self-protection (81.8%; *n* = 153) and protecting others and/or patients (75.9%; *n* = 142) were the most frequently cited reasons to accept the vaccine.

### Opinions and level of social responsibility regarding vaccination

In order to understand the opinion of healthcare professionals on vaccines against COVID-19, the following investigations were conducted. With regard to the latter, 68.3% (*n* = 151) of respondents considered these vaccines to be useful, 63.3% (*n* = 140) assumed that they were effective, and 63.3% (*n* = 140) indicated that these vaccines were safe. Furthermore, 69.7% (*n* = 154) of the interviewees felt that the vaccines were well accepted by healthcare workers. With an additional aim of examining the degree of social responsibility among our healthcare professionals, the following questions were posed: ‘It is my social responsibility to ensure that I am properly protected/vaccinated against COVID-19’; ‘It is a social responsibility for everyone to be vaccinated, unless there are contraindications’. We note that 78.7% (*n* = 174) of healthcare professionals expressed their agreement with the first suggestion and 61.1% (*n* = 135) with the second.

### Overview of the psychological aspects of vaccination

In terms of psychological aspects of vaccination, the scales used to measure the severity of anxiety, perceived stress and insomnia reported that 8.6% (*n* = 19) of participants had mild or moderate to severe anxiety, 51.6% (*n* = 114) had moderate to high stress and 14.5% (*n* = 32) had moderate to severe insomnia.

Table 1, Table 2 and Table 3 summarise the descriptive results of data distribution in our sample.

**TABLE 1:** Sociodemographic characteristics of healthcare workers (*N* = 221).

Variables	*N*	%	Median	Interquartile range
**Gender**				
Female	151	68.3	-	-
Male	70	31.7	-	-
**Age† (years)**	-	-	25.5	30, 34.5
**Marital status**				
Single	121	54.8	-	-
Married	100	45.2	-	-
**Profession**				
Nurse	110	49.8	-	-
Doctor	75	33.9	-	-
Healthcare technician	36	16.3	-	-
**Workplace (hospital centre)**				
University hospital centre	149	67.4	-	-
Provencial hospital centre	56	25.3	-	-
Healthcare centre	16	7.2	-	-
**Seniority† (years)**	-	-	4	1, 8
**Chronic diseases**	-	-	-	-
No	140	63.3	-	-
Yes	81	36.7	-	-
**Seasonal flu vaccination**				
No	170	76.9	-	-
Yes	51	23.1	-	-
**Information on COVID-19**				
Yes	217	98.2	-	-
No	4	1.8	-	-
**Concern about contracting the virus**				
Extremely	26	11.8	-	-
Moderately	51	23.1	-	-
A little	75	33.9	-	-
Not at all	69	31.2	-	-
**Previous COVID-19**				
Yes	163	73.8	-	-
No	58	26.2	-	-
**Compliance with sanitary measures**				
Always	34	15.4	-	-
Often	71	32.1	-	-
Sometimes	76	34.4	-	-
Rarely	28	12.7	-	-
Never	12	5.7	-	-

COVID-19, coronavirus disease 2019.

† , Variable expressed as median (Interquartile range).

**TABLE 2:** Vaccination profile and perception of coronavirus disease 2019 vaccination (*N* = 221).

Variables	*N*	%
**Vaccination profile (1st and 2nd doses)**		
Yes	187	84.6
No	34	15.4
**Vaccination profile (3rd dose)**		
Yes	82	43.9
No	80	42.8
Planned for the next few days	25	13.4
**Hesitation regarding the 3rd dose**		
Yes	58	54.2
No	49	45.8
**Side effects**		
Mild to moderate (for example: Fatigue, Headache, Fever, Nausea)	111	59.4
Severe (for example: Severe Allergic Reaction, Myocarditis, Pericarditis)	0	0.0
No side effects	76	40.6
**Refusal or hesitation reasons**		
Fear of eventual side effects	25	73.5
Vaccination is not necessary	16	47.1
The development time for the vaccines was short	15	44.1
Vaccine is not very effective	13	38.2
Others	8	23.5
**Acceptance reasons**		
Self-protection	153	81.8
Protecting others	142	75.9
Increased risk of infection	99	52.9
Recommendation from the health care community and/or national guidelines	79	42.2
Obligation to do so	75	40.1
Vaccine accessibility	61	32.6
To prevent absenteeism	21	11.2
Others	31	16.6
**Vaccination perception (1st and 2nd dose)**		
Positive perception	172	77.8
Negative perception	49	22.2
**Vaccination perception (3rd dose)**		
Positive perception	96	43.4
Negative perception	125	56.6

**TABLE 3:** Opinions and level of social responsibility regarding vaccination (*N* = 221).

Variables	*N*	%
**Vaccines recommended by public health authorities are useful**		
I agree	151	68.3
I disagree	41	18.6
I do not know	29	13.1
**Vaccines recommended by public health authorities are effective**		
I agree	140	63.3
I disagree	51	23.1
I do not know	30	13.6
**Vaccines recommended by public health authorities are safe**		
I agree	140	63.3
I disagree	49	22.2
I do not know	32	14.5
**Vaccines recommended by the public health authorities have been accepted by healthcare workers**		
I agree	154	69.7
I disagree	45	20.4
I do not know	22	10.0
**Confidence in pharmaceutical companies**		
Yes	79	35.7
No	44	19.9
No comment	98	44.3
**Vaccination recommendation: Before receiving the vaccine**		
Yes	144	77.0
No	43	23.0
**Vaccination recommendation: After receiving the vaccine**		
Yes	131	70.1
No	56	29.9
**It is my social responsibility to ensure that I am properly protected/vaccinated against COVID-19**		
I agree	174	78.7
I disagree	28	12.7
No comment	19	8.6
**It is a social responsibility for everyone to be vaccinated, unless there are contraindications**		
I agree	135	61.1
I disagree	66	29.9
No comment	20	9.0

COVID-19, coronavirus disease 2019.

### Factors of stress related to vaccination in emergency situations

The analysis of participants’ findings indicates that females were more susceptible to vaccine-related stress than males (57% vs. 40%, *p* = 0.019). Additionally, stressed subjects tended to be older than 25.5 years (56.0% vs. 38.2 %, *p* = 0.02). Participants who presented with a higher level of stress were mainly nurses (53.6%, *p* = 0.4) and unmarried participants (53.7%, *p* = 0.4). Yet, the observed differences are not sufficiently significant. On the other hand, healthcare professionals based in university hospital centre were significantly less stressed than those working in regional and/or provincial hospital centre or primary healthcare centre (43.6% vs. 64.3% vs. 81.3%, *p* = 0.001).

The relationship between healthcare professionals and the SARS-CoV-2 virus was marked by a significant level of stress, and this was true, in the group with a high level of fear towards the virus (69.2%, *p* = 0.01). Levels of stress decreased successively with the level of fear, and participants with no fear of contracting the virus were classified as the less stressed (36.2%). In the same way, the stress levels were higher among respondents who complied with sanitary measures than among those who did not. However, the difference was not significant (*p* = 0.1). In addition, participants with a previous COVID-19 infection expressed a higher level of stress than those who had not been contaminated (55.8% vs. 39.7%, *p* = 0.03).

With regard to vaccination profile, healthcare professionals who received their first two doses were declared to be more stressed than non-vaccinated professionals (*p* = 0.09). Furthermore, recipients of a third dose, were found to experience a higher level of perceived stress than non-recipients, or those who were interested in a third dose of the COVID-19 vaccine (*p* = 0.01). We note that participants who were reticent about the booster dose, were significantly more stressed (62.1%, *p* = 0.009).

The acceptance reasons, for which differences were significant, with the least stress were: vaccine accessibility (*p* = 0.01), preventing absenteeism (*p* = 0.04), and vaccine recommendation from the healthcare community and/or national guidelines (*p* = 0.09). In the same way, the proportion of healthcare professionals who declined to be vaccinated and who considered the vaccine development time was brief expressed significantly less stress than the others (*p* = 0.052).

Based on the perception section, respondents with a positive attitude towards the first two doses of the vaccine expressed a higher level of stress than those with a negative perception. On the other hand, the proportion of healthcare professionals with a negative perception of the booster dose was higher in the category of stressed respondents. Nevertheless, the differences observed are largely non-significant (*p* = 0.6, *p* = 0.8).

No significant association was observed between the degree of social responsibility of healthcare professionals and stress regarding vaccination.

### Binary logistic regression analysis findings

The binary logistic regression results indicated that females were 1.98 times more susceptible to experiencing vaccination stress (95% confidence interval [CI]: [1.067–3.666], *p* = 0.03). Furthermore, vaccination profile (OR: 2.596, 95% CI: [1.150–5.863], *p* = 0.02), having a previous COVID-19 infection (OR: 2.036, 95% CI: [1.060–3.908], *p* = 0.03), and accepting the vaccine for any reason other than its accessibility (OR: 2.037, 95% CI: [1.039–3.994], *p* = 0.03), were significantly associated with stress. In contrast, healthcare professionals based at the university hospital had a significant lower stress level (OR: 0.201, 95% CI: [0.052–0.768], *p* = 0.01).

Table 4 and Table 5 summarise the findings of the binary logistic regression analysis of stress-related risk factors in relation to vaccination.

**TABLE 4:** Univariate and multivariate analysis of factors influencing stress regarding coronavirus disease 2019 vaccination (*N* = 221).

Variables	Stress present		*p*	OR	95% CI	*p*
	*n*	%				
**Gender**	-	-	0.019	1.977	1.067–3.666	0.030
Female	86	57.0	-	-	-	-
Male	28	40.0	-	-	-	-
**Age (years)**		-	0.020	-	-	--
< 25.5	21	38.2	-	-	-	-
> 25.5	93	56.0	-	-	-	-
**Marital status**	-	-	0.400	-	-	--
Single	65	53.7	-	-	-	-
Married	49	22.2	-	-	-	-
**Profession**	-	-	0.400	-	-	--
Nurse	59	53.6	-	-	-	-
Doctor	40	53.3	-	-	-	-
Healthcare technician	15	41.7	-	-	-	-
**Workplace (hospital centre)**	-	-	0.001	-	-	-
University hospital centre	65	43.6	-	0.201	0.052–0.768	0.019
Regional/Provincial hospital centre	36	64.3	-	0.446	0.106–1.877	0.271
Primary healthcare centre	13	81.3	-	1.000	-	-
**Seniority (years)**	-	-	0.400	-	-	--
< 4	68	54.0	-	-	-	-
> 4	46	48.4	-	-	-	-
**Chronic diseases**	-	-	0.200	-	-	--
No	68	48.6	-	-	-	-
Yes	46	56.8	-	-	-	-
**Seasonal flu vaccination**	-	-	0.300	-	-	--
No	85	50.0	-	-	-	-
Yes	29	56.9	-	-	-	-
**Information on COVID-19**	-	-	0.300	-	-	--
Yes	111	51.2	-	-	-	-
No	3	75.0	-	-	-	-
**Concern about contracting the virus**	-	-	0.010	-	-	--
Extremely	18	69.2	-	-	-	-
Moderately	30	58.8	-	-	-	-
A little	41	54.7	-	-	-	-
Not at all	25	36.2	-	-	-	-
**Previous COVID-19**	-	-	0.030	2.036	1.060–3.908	0.033
Yes	91	55.8	-	-	-	-
No	23	39.7	-	-	-	-
**Vaccination profile (1st and 2nd doses)**	-	-	0.090	2.596	1.150–5.863	0.022
Yes	101	54.0	-	-	-	-
No	13	38.2	-	-	-	-
**Vaccination profile (3rd dose)**	-	-	0.010	-	-	--
Yes	47	58.8	-	-	-	-
No	47	57.3	-	-	-	-
Planned for the next few days	7	28.0	-	-	-	-
**Hesitation regarding the 3rd dose**	-	-	0.009	-	-	--
Yes	36	62.1	-	-	-	-
No	18	36.7	-	-	-	-
**Vaccination perception**	-	-	0.600	-	-	--
Positive perception	90	52.3	-	-	-	-
Negative perception	24	49.0	-	-	-	-
**Confidence in pharmaceutical companies**	-	-	0.300	-	-	--
Yes	36	45.6	-	-	-	-
No	25	56.8	-	-	-	-
No comment	53	54.1	-	-	-	-

OR, odds ratio; CI, confidence interval; COVID-19, coronavirus disease 2019.

**TABLE 5:** Univariate and multivariate analysis of acceptance and refusal reasons influencing stress regarding coronavirus disease 2019 vaccination.

Variables	Reasons	Yes		No		*p*	OR	CI 95%	*p*
		*n*	%	*n*	%				
	Self-protection	80	52.3	21	61.8	0.316	-	-	--
	Protecting others	75	52.8	26	57.8	0.561	-	-	--
	Increased risk of infection	50	50.5	51	58.0	0.308	-	-	--
**Acceptance reasons (*n* = 187)**	Recommendation from the health care community/national guidelines	37	46.8	64	59.3	0.090	-	-	--
	Obligation to do so	37	49.3	64	57.1	0.294	-	-	--
	Vaccine accessibility*	25	41.0	76	60.3	0.010	2.037	1.039–3.994	0.038
	To prevent absenteeism	7	33.3	94	56.6	0.040	-	-	--
	Others	15	48.4	86	55.1	0.492	-	-	--
	Fear of eventual side effects	9	36.0	4	44.4	0.700	-	-	--
	Vaccination is not necessary	4	25.0	9	50.0	0.100	-	-	--
**Refusal or hesitation reasons (*n* = 34)**	The development time for the vaccines was short	3	20.0	10	52.6	0.052	-	-	--
	Vaccine is not very effective	3	23.1	10	47.6	0.200	-	-	--
	Others	2	25.0	11	42.3	0.300	-	-	--

OR, odds ratio; CI, confidence interval; COVID-19, coronavirus disease 2019.

* , In the multivariate analysis, we took the answer “Yes” as a reference.

## Discussion

The decision to vaccinate is a behavioural intention, based on a series of behavioural and social factors. According to Brewer and his collaborators, there are two main possible motivational factors for vaccination.^1^ The first barrier concerns individuals’ evaluation of current disease rates and their confidence level in immunisation (opinion and feelings). On the other hand, social variables such as vaccination recommendation by a healthcare worker, familial support, etc. represent the second actor in this set of decision. A third challenge faces the practical aspect of such motivation, that is, the availability and accessibility of vaccines.

We carried out this survey to understand the interactions between various factors influencing immunisation compliance. The component related to vaccination confidence among healthcare professionals was examined, as the quality of care depends on the health of the caregivers.

Gender, rather than sex, was considered in this study in order to determine the socio-cognitive factors influencing healthcare professionals’ attitudes and behaviour.^19^

According to the analysis of the findings, gender had a significant impact on vaccination stress level. Indeed, female healthcare workers developed more significant stress (*p* = 0.019). Such a divergence in terms of mental health between the two genders has been observed in the literature.^20,21,22,23^ An investigation of Bangladeshi healthcare professionals revealed that females presented a higher mental health problem than males.^24^ In the same study, vaccinated female healthcare providers exhibited anxiety levels that were 2.17 times greater.^24^ In a separate report, female participants indicated that they were more susceptible to depression, anxiety and post-traumatic stress disorder, in both the vaccinated and non-vaccinated groups.^21^ Several explanations have been proposed to account for the observed gender differences in stress regarding vaccination. On average, females are often socialised to value a more ‘natural’ lifestyle, one that is perceived as being closely connected to the body.^12^ Vaccination, in this context, may be seen as a technological intervention that disrupts the ‘natural’ functioning of the body, leading to greater hesitancy or stress among women. Additionally, females may be more concerned about the potential risks of vaccination, particularly in relation to pregnancy and their children’s health.^12^ Regarding physiology, females are more exposed to adverse effects following vaccination than males.^25,26^ It appears that the physiological and psychological structure of females makes them particularly vulnerable to disruptive and stressful events, resulting in increased levels of stress.^21^ Consequently, they are more concerned about the safety and efficacy of vaccines than males. Such behaviour could contribute to declining vaccination rates among females.^27^ Given the mental health problems faced by this population, it is therefore wise to consider gender-specific approaches to offering them targeted awareness-raising programmes. This initiative aims to encourage participants to get vaccinated while preserving their confidence and mental health regarding vaccination.^21^ These factors, including cultural, social, and physiological considerations, contribute to the higher levels of stress regarding vaccination observed among female participants. A deeper understanding of these factors is essential to developing targeted interventions that can address and mitigate these concerns.

Age was another socio-demographic variable significantly associated with vaccination stress. From our analytical data, older healthcare professionals included in this study tended to be more stressed than younger ones. Previous studies, with divergent results, had pointed to the relationship between age and vaccination behaviour.^21,23,28,29^ This suggests that older healthcare professionals are more concerned about their safety where new vaccines are involved.^29^ Another possibility is that older healthcare employees are more experienced and have more seniority and are therefore more attentive in emergency situations.^30^

Additional demographic variables were identified in this study that seem to be associated with psychological aspects of immunisation behaviour, such as occupation and workplace. Indeed, while not significant, laboratory technicians were the least stressed group compared with doctors and nurses. This observation is in line with previous surveys, in which nurses and doctors were more reticent about vaccines.^23,31,32^ Yet, these studies highlighted a difference in attitudes even between doctors and nurses, with a positive trend among doctors. This might be explained through the fact that doctors have been trained to accept concessions when the benefits outweigh the risks.^15^ Instead, nurses may often decide to leave their posts rather than be vaccinated.^32^ We suggest that the nature of the work performed by laboratory technicians accustoms them to dealing appropriately with biological fluids. They have therefore developed a particular behavioural pattern that is quite different from that of doctors and nurses, who tend to work in direct contact with patients.

On the other hand, healthcare professionals practicing in the university hospital centre presented a significant lower stress level, compared with that developed within the regional/provincial centres and the primary healthcare centres. Since the start of the pandemic in Morocco, the majority of COVID-19 patients have been hospitalised in university hospitals. This allowed healthcare professionals in the university hospitals to become more accustomed to COVID-19 infections, while reducing their concerns about the virus. This practice may have allowed them to familiarise with sanitary measures to protect themselves, including vaccination, and therefore to develop a positive vaccination behaviour. This highlights the need to offer psychological support to healthcare professionals in other centres to improve immunisation coverage.

Other interesting outcomes identified in this study include the impact of concern about the SARS-CoV-2 virus on the level of stress associated with vaccination. Such an association has previously been reported in previous studies, emphasising the effect of complacency as one of five psychological antecedents susceptible to influence an individual’s decision to vaccinate.^33,34,35^ Overall, complacency regarding diseases prevented by vaccination could lead to a decline in vaccination coverage and a further increase in their propagation. Our results indicate that stress levels were significantly higher in healthcare workers who were concerned about infection. A study conducted among populations in Arabic-speaking countries revealed that being a healthcare professional is a protective factor against complacency (OR: 0.512; 95% CI: 0.387–0.678).^33^ Thus, healthcare professionals might be more aware of emergency situations, whether concerning the pandemic or new vaccines.

Results from this study have also highlighted the impact of a previous history of COVID-19 on the level of stress associated with vaccination. Indeed, participants with a previous COVID-19 infection expressed a more remarkable stress. These results are consistent with a previous study conducted among healthcare professionals, where the cumulative anxiety score was significantly higher in the group of healthcare providers with a positive history of COVID-19 infection.^36^ According to previous data, having a previous COVID-19 infection improves the awareness of vaccine benefits and, therefore, increases its acceptability.^37,38,39,40^ In contrast, another study indicated that non-contaminated individuals are quite prepared to accept vaccination.^39^ On the other hand, the study conducted by Abdou and collaborators reported a detrimental impact of COVID-19 history on immunisation coverage, suggesting an increase in the phycological component associated with complacency (OR: 1.556; 95% CI: 1.171–2.068).^33^ It seems possible that the healthcare professionals in our sample are more concerned about their safety and therefore are more anxious and apprehensive about vaccination.

The impact of the vaccination profile on stress levels towards vaccination was evaluated. According to the results, vaccinated healthcare professionals had a slightly higher level of stress than non-immunised ones (*p* = 0.09). The difference was more significant for the booster dose, and the vaccinated group remained the most stressed (*p* = 0.01), with a higher stress level among the reluctant group (*p* = 0.009). We note that vaccination rate went from 84.6% for the first two doses, to 43.9% for the booster dose, with a decline of more than 50%. Such an outcome was not expected from healthcare professionals, given their profession and experience and as they assumed that the vaccines recommended by the public health authorities were useful, effective and safe. These observations are consistent with the findings of a previous study carried out among young adults in Morocco, where the percentage of stressed participants within the group vaccinated with the third dose was significantly higher.^29^ Even if the stress level was noticeably higher among vaccinated participants, the immunisation acceptance rate observed in our study remains better than that reported in Alalawi et al.’s meta-analysis.^41^ Hence, it seems that both the pressing nature of the pandemic and the rapid distribution of SARS-CoV-2 vaccines prompted the emergence of such stress regarding vaccination. Despite this, neither acceptance of the vaccine nor healthcare professionals’ attitudes towards immunisation have changed. Thus, further studies are required in order to better understand the role of emergency situations on the relationship between vaccine uptake and vaccine stress.

Furthermore, no significant association was reported between immunisation perception and stress towards SARS-CoV-2 vaccines. Nevertheless, our findings indicate that stress was higher among healthcare workers with a positive perception of vaccination. Previous research has revealed the link between the psychology of vaccination behaviour, as well as vaccine perception and acceptance.^29,36^ Indeed, these results are consistent with the findings of Alalawi and his collaborators, where 66% of healthcare workers included in their survey had presented different states of anxiety and depression. The same study also highlighted a positive perception and knowledge of immunisation despite the low vaccination rate.^36^ On the other hand, a systematic review designed to investigate healthcare professionals’ behaviour towards the COVID-19 vaccine and the associated factors, emphasised the impact of psychological factors on vaccination perception and uptake. Thus, the study of psychological antecedents of vaccination using the 5C scale^42^ revealed that the highest rates of vaccine acceptance were observed among subjects with a high level of self-confidence and sense of collective responsibility, a low level of complacency, and a low level of benefit and risk evaluation.^43,44^ Another study suggests that the occurrence of post-traumatic stress symptoms could have a negative impact on vaccination decision.^45^ Indeed, it appears that the stress is susceptible to elicit negative cognitive responses, notably negative attitudes and behavioural responses.^46^ Hence, our results indicate a high level of stress associated with a positive perception, which might be explained through the fact that while the participants included in the current survey presented a positive perception, they were systematically concerned about the personal aspect of their safety. The non-significance of our results could be attributed to the period when the survey was performed. Indeed, prior studies have demonstrated that vaccine intent changes over time.^47,48^

The data also highlighted a significantly lower level of stress, among participants who agreed to be vaccinated because of its accessibility (*p* = 0.01), to avoid absenteeism (*p* = 0.04) and because the vaccine is recommended by the healthcare community and/or national guidelines (*p* = 0.09). A survey of Moroccan healthcare professionals identified self-protection, others and patients protection as the most cited reasons for vaccine acceptance.^49^ Additional studies have reported that among factors that motivated healthcare professionals to get vaccinated were self-protection as well as family, patient and community protection.^50,51^ Our descriptive results agreed with the conclusions of the above-mentioned studies. Thus, in terms of stress level, the findings indicate that our respondents seem more willing to accept the vaccine because of its accessibility. It is possible that the availability and accessibility of such vaccines would have a double beneficial effect on healthcare professionals’ immunisation behaviour, as they would experience positive emotions while accepting such action.^29^ Accordingly, vaccines should not only be produced and available, but also be accessible to everyone. Hence, it is appropriate to assert that broadening access to vaccination could profoundly alter the situation.

Vaccine recommendation by the healthcare community and/or national guidelines appeared among the reasons for acceptance with the least association with stress. Previous investigations indicate that healthcare workers are more motivated to vaccinate when the vaccine is recommended by doctors as well as healthcare and public health authorities.^49,52^ Therefore, this finding highlights the considerable confidence accorded to national authorities, directives and decision-makers, which makes these vaccines highly acceptable among healthcare professionals.

Participants who considered that immunisation could contribute to prevent absenteeism also expressed a lower level of stress. This result is in line with the conclusions of a critical review, which suggested that healthcare professionals have a more altruistic attitude to vaccination.^51^ It may be that their appreciation of the possibility of contracting the disease and its potential seriousness, balanced with their high level of responsibility expressed towards their patients, allows them to assess the concept of vaccination as an effective tool to prevent absenteeism.

In the same way, the refusal reason with the least association with stress was the rapid development and approval of COVID-19 vaccines. Previous studies indicated that fear of potential side effects and complacency were cited as the main reasons for hesitation and/or refusal regarding vaccination.^33,49^ The accelerated development of vaccines against COVID-19, because of the emergency nature of the pandemic, has generated a number of doubts, including those concerning various adverse effects. Such concerns might prompt negative cognitive responses reflected in a definite unwillingness to accept vaccination.

### Limitations of the study

We are conscious of the limitations of our investigation as the cross-sectional nature of the study design may not allow the establishment of causality between stress and its relationship with vaccine willingness and actual future vaccination. Furthermore, snowball sampling, while effective for reaching hard-to-access populations, has notable limitations. It may introduce sampling bias because of its reliance on participants recruiting acquaintances, potentially resulting in a sample that lacks diversity and does not fully represent the broader population. This method can also lead to a homogeneous sample in terms of social or professional characteristics, which may limit generalisability. In addressing the limitations of the psychological scales used to measure anxiety and stress in our study, it is important to acknowledge that these tools, while widely used, have certain constraints that may impact the results. For instance, the validity and reliability of these scales can vary, potentially influencing the consistency and generalisability of the findings. The measurement tools used in our study might not fully capture the complexity of stress and anxiety, leading to potential underestimation or overestimation of these psychological states. Furthermore, the timing of the study could also lead to errors, as the perception of vaccination could change with the pandemic health situation.

Nevertheless, given the absence of studies examining the potential factors associated with vaccination stress among Moroccan healthcare professionals, the findings of the present study remain of considerable significance.

## Conclusion

This survey revealed that Moroccan healthcare professionals exhibited a high level of vaccine acceptance and positive perceptions, especially among those who were vaccinated, despite experiencing relatively high levels of stress related to immunisation. It challenges the common misconception that healthcare workers, because of their scientific and medical training, automatically hold positive attitudes towards vaccinations. These findings underscore the need for governments and policymakers to develop targeted interventions that not only enhance healthcare workers’ awareness and knowledge about the value of vaccination but also address their mental health and stress levels associated with immunisation.

To improve vaccination strategies among healthcare workers, public health policies should include comprehensive educational programs that address both the benefits of vaccination and strategies to mitigate stress and anxiety related to immunisation. Furthermore, there is a need for institutional support systems to promote a positive vaccination culture within healthcare settings.

Future research should explore the specific cultural, social and psychological factors contributing to stress towards vaccination among healthcare professionals. To advance our understanding of the relationship between vaccination behaviour and stress regarding immunisation, future longitudinal research should be conducted to elucidate the causal mechanisms and temporal dynamics of this interaction. Additionally, studies could assess the effectiveness of targeted interventions in reducing stress and improving vaccination uptake and attitudes. By addressing these broader implications, our findings provide a foundation for informed public health strategies that support healthcare workers’ well-being and promote higher vaccination rates within this critical group.
